# Molecular and Morphological Characterization of the Entomopathogenic Nematode *Oscheius cyrus* (Nematoda: Rhabditidae) and Molecular Variability of *Heterorhabditis bacteriophora* from Georgia (Caucasus)

**DOI:** 10.3390/biology14050512

**Published:** 2025-05-07

**Authors:** Oleg Gorgadze, Elena Fanelli, Alessio Vovlas, Alberto Troccoli, Eustachio Tarasco, Francesca De Luca

**Affiliations:** 1 Institute of Zoology, Ilia State University, Tbilisi 0162, Georgia; oleg.gorgadze@iliauni.edu.ge; 2 Institute for Sustainable Plant Protection-CNR, Via Amendola 122/D, 70126 Bari, Italy; elena.fanelli@cnr.it (E.F.); alessiovovlas@cnr.it (A.V.); alberto.troccoli@cnr.it (A.T.); 3 Department of Soil, Plant and Food Sciences, Section of Entomology and Zoology, University of Bari “A. Moro”, Via G. Amendola, 165/A, 70126 Bari, Italy; eustachio.tarasco@uniba.it

**Keywords:** *Insectivorus* group, mitochondrial COI, molecular, morphometrics, *Oscheius*, phylogeny, taxonomy

## Abstract

Surveys were conducted in the village of Shamgona in the Samegrelo Region of West Georgia, and two nematode species belonging to the *Oscheius* genus and a population of *Heterorhabditis bacteriophora* were isolated. Integrative diagnosis, combining morphology, PCA, sequencing and phylogeny, was carried for *Oscheius* sp. from the soil of hazelnut orchard. This population shows high similarity at morphological and morphometrical levels with *O. cyrus* from Iran, and the main distinctive features with *O. cyrus* from Iran are the presence of eight lateral lines and males. Sequence and phylogenetic analyses by using ITS, D2–D3 expansion domains of the 28S rRNA gene, the partial 18S rRNA gene, and the mitochondrial COI confirmed the identification as *O. cyrus.* A second population of *Oscheius* sp. and *H. bacteriophora* was characterized only at the molecular and phylogenetic level. The phylogenetic analyses, based on ITS and D2–D3 expansion domains, revealed the occurrence of *O. insectivorus* in Georgia grouping with *O. insectivorus* sequences with high support. The phylogenetic trees of *H. bacteriophora*, based on D2–D3 expansion domains, ITS, the partial 18S rRNA gene, and mitochondrial COI confirmed the high variability among geographical populations. The present study reports on the occurrence of two species of *Oscheius* in Georgia for the first time.

## Simple Summary

Entomopathogenic nematodes are useful biocontrol agents (BCA) used in the management of insects worldwide. Two genera of entomopathogenic nematodes, *Heterorhabditis* and *Steinernema*, are well known as BCA agents. Recently, the genus *Oscheius* has received attention from researchers, as several species display entomopathogenic ability. During a recent soil survey for entomopathogenic nematodes in Georgia, two *Oscheius* species and a population of *Heterorhabditis bacteriophora* were recovered through the Galleria-bait method. An integrative approach, combining PCA, morphological, molecular, and phylogenetic analyses, was applied to identify the two *Oscheius* species. Our results report the new occurrence of two species of *Oscheius* in Georgia and their potential to be used as BCAs.

## 1. Introduction

Entomopathogenic nematodes (EPNs) are soil-dwelling nematodes occupying diverse habitats. They are powerful biological control agents (BCAs) against insect pests of various crops [1,2] and are distributed worldwide [3,4,5]. Two genera, *Heterorhabditis* and *Steinernema*, are well-known EPNs and used as BCAs. Several members of the Rhabditidae family have been reported as entomopathogenic nematodes [5,6,7,8,9]. The genus *Oscheius* Andrassy (1976) [10] has gained attention by researchers in recent years as several species display entomopathogenic ability [5,6,7,8,9,11] or scavenging behavior [12,13]. This genus consists of two group of species: *Insectivorus* group and *Dolichura* group. The first group (*Insectivorus*) has a leptoderan bursa, while the second group (*Dolichura*) has a peloderan bursa [6,7,14,15]. In order to improve their biocontrol potential, it is important to isolate local strains or to identify species adapted to local climatic conditions. Accurate identification of the specific strain is also important for effective control, as the systematics of several species of the genus are poorly described with no molecular support; thus, misidentifications are reported in the literature and in sequences in GenBank. Currently, several EPN species are reported from Georgia, namely, *S*. *georgica* [16], *S. disparica* [17], *S. gurgistana* [18], *S. thesami* [19,20], *S. borjomiensis* [21], *Pelodera strongyloides* [22], and *Phasmarhabditis thesamica* [23], but no *Oscheius* species were reported so far. During a field survey for entomopathogenic nematodes in two regions of Georgia, mixed populations of nematodes were recovered and investigated for the ability to kill *Galleria mellonella*. In both regions, two nematode species belonging to *Oscheius* genus and one species to *Heterorhabditis bacteriophora* were present. Interestingly, the abundant nematode population from the soil of hazelnut orchard in the village of Shamgona, in the Samegrelo Region of West Georgia, was characterized at morphological, multivariate analysis, at the molecular and phylogenetic level, and it was identified as *O. cyrus* [24]. The other two populations were characterized only at the molecular and phylogenetic level and identified as *O. insectivorus* and *H. bacteriophora*. Entomopathogenecity tests were also conducted using *G. mellonella* and *Tenebrio molitor* L. (Coleoptera: Tenebrionidae) for all three species. Based on phylogenetic results, the status of few *Oscheius* species is also discussed.

*Oscheius cyrus* was first described from soil samples collected from forest heights in Iran. This species was described at the morphological and molecular level, also providing COI sequences. In addition, *O. cyrus* was also able to suppress *G. mellonella*. Little is known about the morphometric and molecular variability of *O. insectivorus*. Recently, [25] performed both the morphological and molecular identification of a population of *O. insectivorus* from Iran, including the sequencing and phylogenetic analyses and the evaluation of the pathogenicity of the Iranian isolate.

## 2. Materials and Methods

### 2.1. Soil Sampling and Nematode Recovery

During surveys conducted in Spring 2018, sixty-eight soil samples were collected, and fourteen out of sixty-eight soil samples were positive for EPNs. Twelve samples contained *O. cyrus*, with two of them containing *O. cyrus* and *O. insectivorus*, and two containing *H. bacteriophora*. Two positive samples for EPNs were characterized in the present paper, one from hazelnut orchards (42°31′09′’ N; 41°46′10′’ E), in the village of Shamgona in Samegrelo Region of West Georgia, 60 m above sea level, and the second one was from the municipality of Borjomi, in the territory of the Borjomi-Kharagauli reserve. Soil samples were taken by using a cross-sampling design method, to a depth of about 15 cm. Each soil sample (approximately 250–300 g) was placed in a polyethylene bag, sprayed with water, appropriately labeled and sent to the laboratory for further analysis.

Soil samples were tested for the presence of entomopathogenic nematodes using the *Galleria* bait method [26]. Sixty-eight plastic containers (16 × 7 × 8 cm) were used, and 4–5 *G. mellonella* larvae were placed in each container. After 5 days of incubation, insect cadavers were transferred to a modified White’s trap [27], and the emerged nematodes were recovered. When mixed populations were present, the different populations were separated based on identical morphological characters and used separately to infect *G. mellonella*.

Extracted nematodes from hazelnut orchards were morphologically and morphometrically studied in Tbilisi, in the laboratory of entomopathogens of the Institute of Zoology of the Ilia State University (Georgia), whereas molecular analyses of both samples were carried out at the Institute for Sustainable Plant Protection in Bari, Italy.

### 2.2. Morphological and Morphometric Identification of Oscheius cyrus

Temporary mounts were made for infective juveniles (27 specimens) and adult nematodes of both sexes (20 females and 20 males). Nematodes were examined by gently heating in Ringer’s solution at 60 °C and then mounted in a temporary water agar slide [28,29] for preliminary observation and pictures. Permanent slides were obtained by fixing nematodes in hot TAF [30] and processed into anhydrous glycerol according to the study of [31]. Measurements, drawings, and photographs of specimens were taken using a light microscope Motic^®^-DMB1 (Xiamen, China) equipped with a full set of objectives and a digital video camera Genius G-Shot DV 1110 (KYE Systems Co., Taiwan, China). Slide, coverslips were supported by glass rods to avoid flattening of specimens. Morphometric characters were based on the main diagnostic characters reported by [32]. All measurements were taken in micrometers (µm) and expressed as mean ± standard deviation.

### 2.3. Multivariate Morphometric Analysis

To evaluate the degree of morphological variations within *Oscheius* species, including those of the present study, a Principal Component Analysis (PCA) of the different morphological traits was conducted [33,34]. PCA was carried out in XLSTAT [35]. Measurements were obtained from the literature, using the mean values for each population. Measurements were normalized through XLSTAT prior to their analysis. The PCA was carried out using nine diagnostic characters: body length (L), ‘de Man’s indices’ (a, b, c, c’), percentage distance from anterior end to vulva/body length (V), body diameter at mid-body (MDB), tail length (T), and anal body diameter (ABD). The score values for the first two components were determined to form a two-dimensional plot (PC1 and PC2) of each population based on factor loadings given by the software.

### 2.4. Entomopathogenicity of Oscheius cyrus

To assess entomopathogenicity of *O. cyrus* from hazelnut, instar larvae of *G. mellonella* (Lepidoptera: Galleridae) and instar larvae of mealworm *T. molitor* were added in Petri dishes [36]. Two layers of filter papers were placed in each Petri dish, 9 cm in diameter, with ten experimental insect larvae. Three-thousand infective nematode larvae (i.e., 300 nematodes/insect) were inoculated into each dish. Control larvae were treated with pure water. Petri dishes were stored at 25 °C in the dark with a fully randomized design. Five replicates were carried out, and the experiments were repeated twice. Insect mortality was assessed every 24 h [37]. Half of dead larvae were dissected to evaluate the presence of nematodes. The rest of insect cadavers were transferred to White’s traps to check nematode emergence. Emerged nematodes were stored in a refrigerator at 5–6 °C for further morphological identification. Several nematodes were stored in 70% ethanol and then sent to the Institute for Sustainable Plant Protection in Bari (Italy) for molecular studies.

### 2.5. Molecular Identification

DNA was extracted from individual infective juveniles of *O. cyrus*, *O. insectivorus*, and *H. bacteriophora*. Specimens were handpicked and singly placed on a glass slide in the lysis buffer (10 mM of Tris-HCl, pH8.8, 50 mM of KCl, 15 mM of MgCl_2_, 0.1% Triton X100, with 90 µg/mL proteinase K) and then cut into small pieces under a dissecting microscope. The samples were incubated at 65 °C for 1 h and then at 95 °C for 15 min to deactivate the proteinase K. The PCR amplification, cloning, and sequencing protocols were described in detail by the authors of [38]. The following primers were used for the ITS1-5.8S-ITS2 region using the forward primer TW81 (5′-GTTTCCGTAGGTGAACCTGC-3′) and the reverse primer AB28 (5′-ATATGCTTAAGTTCAGCGGGT-3′) [39]; for the D2–D3 expansion segments of 28S rRNA using forward D2A (5′-ACAAGTACCGTGGGGAAAGTTG-3′) and reverse D3B (5′-TCGGAAGGAACCAGCTACTA-3′) [40]; the mitochondrial COI was amplified using COI-F1 (5′-CCTACTATGATTGGTGGTTTTGGTAATTG-3′) and COI-R2 (5′-GTAGCAGCAGTAAAATAAGCACG-3′) [41]; and the 18S rDNA was amplified using the 18SnF (5′-TGGATAACTGTGGTAATTCTAGAGC-3′) and 18SnR (5′-TTACGACTTTTGCCCGGTTC-3′). PCR amplifications were carried out in 100 µL volumes. PCR mix was added to each tube: 10 µL 10×PCR buffer, 2 µL dNTP mixture (10 mM each), 2 µL of each primer (10 mM), 0.25 µL of Taq DNA polymerase (Roche), 73.5 µL of distilled water, and 10 µL of crude DNA. The cycling conditions were as follows: 1 cycle of 94 °C for 7 min., followed by 35 cycles of 94 °C for 50 sec., 55 °C for 50 sec., and 72 °C for 50 sec. The last step was 72 °C for 10 min. PCR products of the ITS containing region, D2–D3 expansion domains of the 28S rRNA gene, the 18S rRNA gene and the partial mitochondrial COI from three individual nematodes were purified for sequencing using the protocol listed by manufacturer of Nucleospin Extract II (Macherey-Nagel, Duren, Germany) gel extraction kit. The ITS, 18S rRNA gene, and the mitochondrial COI were cloned with pGEM-T Vector System II kit (Promega, Madison, WI, USA). Positive clones were sent for sequencing in both directions with the primers given above or M13 forward and M13 reverse primers to Eurofins Genomics (Ebersberg, Germany). Newly obtained sequences were deposited in the GenBank under accession numbers: PV400785- PV400786 for the D2–D3 expansion domains of the 28S rRNA gene, PV428539- PV428540 for the ITS region, PV400801 for 18S rRNA gene of *O.cyrus*; PV400787 for the D2–D3 expansion domains of the 28S rRNA gene, PV428541 for the ITS for *O. insectivorus*; and PV400788 for the D2–D3 expansion domains of the 28S rRNA gene, PV428542 for the ITS, P400802-PV400803 for *H. bacteriophora*.

### 2.6. Phylogenetic Analysis

Phylogenetic analyses were carried out using the new sequences of ITS, 18S rRNA gene, D2–D3 expansion domains of the 28S rDNA and mitochondrial COI along with the corresponding sequences of *Oscheius* present in GenBank by using MAFFT v.7.450 [42]. Sequence alignments were manually edited using BioEdit 7.2.5 [43].

The best-fit model of nucleotide substitution used for the phylogenetic analysis was statistically selected using jModelTest 2.1.10 [44] with the Akaike information criterion. The best evolution models were SYM + G for ITS, HKY + G for 18S, GTR + G for 28S, and COI, and they were included in the Bayesian inference (BI) using Mr.Bayes 3.2.7 [45] for two million generations for all datasets. The Markov chain Monte Carlo (MCMC) method sampled every 100 generations [46] using the 50% majority rule and to estimate posterior probabilities in the phylogenetic trees. Then, the burn-in step was set at 25% of the convergent runs. The output files of the phylogenetic program were visualized and saved with FigTree 1.4.3 [47]. Posterior probabilities (PPs) exceeding 50% are given on the appropriate clades.

## 3. Results

### 3.1. Morphological and Morphometric Observations of Oscheius cyrus

**Figure 1:**
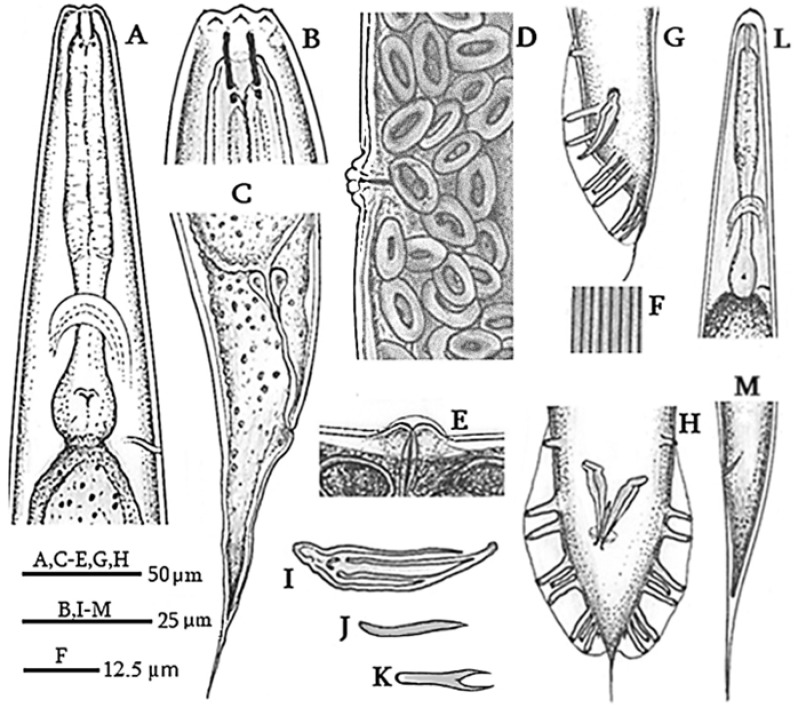
*Oscheius cyrus* line drawings: (**A**) Female pharyngeal region. (**B**) Female anterior end. (**C**) Female tail region in lateral view. (**D**,**E**) Female vulva and posterior genital region. (**F**) Lateral incisures. (**G**,**H**) Male tail, in lateral and ventral view, respectively. (**I**) Male spicules. (**J**,**K**) Male gubernaculum. (**L**) Male pharyngeal region. (**M**) Male tail region in lateral view.

**Figure 2:**
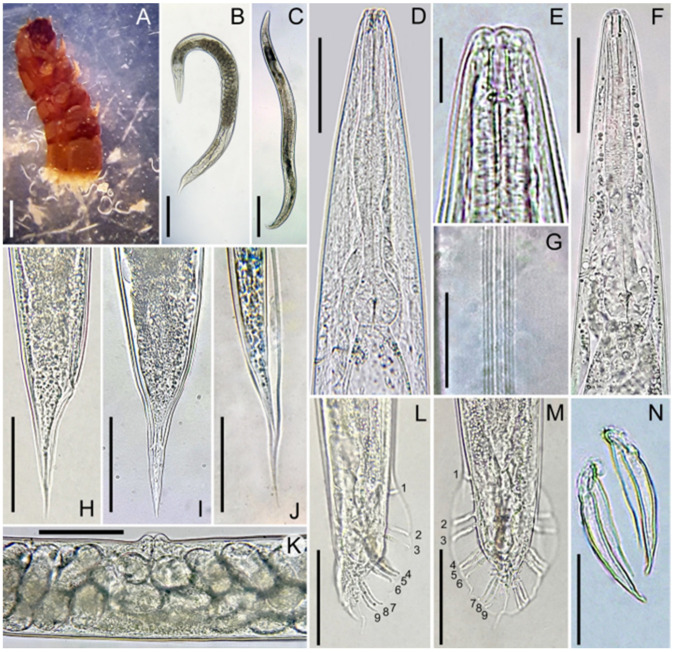
(**A**) Entomopathogenicity bioassay on *Galleria mellonella*. (**B**,**C**) Body habitus of female (**B**) and male (**C**). (**D**) Female pharyngeal region. (**E**) Female anterior end. (**F**) Male pharyngeal region. (**G**) Lateral fields at mid body. (**H**) Female tail in lateral view. (**I**) Female tail in ventral view. (**J**) Tail region of dauer juvenile. (**K**) Vulval region. (**L**) Tail in lateral view (papillae are indicated). (**M**) Tail in ventral view (numbers indicate genital papillae). (**N**) Spicules (Scale bars: (**B**,**C**) = 200 μm; (**D**,**F**,**K**) = 50 μm; (**E**) = 20 μm; (**G**) = 12.5 μm; and (**H**–**J**,**L**–**N**) = 25 μm).

Measurements and Figures are in Table 1 and Figure 1 and Figure 2, respectively.

The female body is almost straight and spindle-shaped, cuticle smooth, 5.2 µm thick at the head and tail, and 2.6 µm thick at mid-body. Amphid pores are located on the lateral lips behind the labial sensillae. The stomata is four times as long as wide with a pharynx cylindrical (Figure 1A) and a basal bulb that is averagely large and developed. The excretory canal is sclerotized in the terminal part. There are lateral fields with eight lines at mid-body (Figure 1F). The reproductive system is didelpho-amphidelphic, bearing, on average, 112 eggs in each uterus. The vulva transverse is located about mid-body. In young females, vulval lips are slightly raised, while, in mature females they are more prominent. A transverse appendage is visible on the vulva of mature females (Figure 1E and Figure 2K). The rectum is about 79.5 µm in length, and the tail is conical and elongated (Figure 1C and Figure 2H,I). Phasmids are in the distal third of the caudal region, visible on the ventral side.

The male body can be straightened by heating. The dead male rarely has a bent tail. Six lips present provided each with a bristle-shaped labial sensillum. There are four cephalic sensillae, one on each sublateral lip. Amphidial diaphragms are elliptical in shape and located on the lateral lips behind the labial sensillae. The body diameter near the labial lips is 15 ± 1 (13–15) µm. Stoma aperture is triangular, with two lips on each side. Stoma is four times as long as the width. The pharynx is well developed and isomorphic. The pharyngeal collar occupies 55% of the stomatic capsule. Procorpus and metacorpus are not clearly differentiated. The basal bulb is rounded, with clearly visible double haustrulum valve plates. The nerve ring is located at the middle of isthmus. Excretory pores are at the level of the basal bulb (Figure 2F). The seminal gland is monodelphic. Spermatocytes are arranged in multiple rows. Spicules are paired and not merged (Figure 1H and Figure 2N), slightly curved ventrally in lateral view. The gubernaculum is thin and flat, often not visible beneath the spicules, and it makes up 41% of spicule length. Bursa leptoderan and open, with nine pairs of papillae (Figure 1G,H and Figure 2L,M), three precloacal and six postcloacal. Pre-cloacal pairs are separated, with a space gap between the second and third pair smaller than that between the first and second pair. The fourth, fifth, and sixth post-cloacal pairs form the first group, while the seventh, eighth, and ninth pairs form the second post-cloacal group. Among them, the third, fourth, sixth, seventh, and ninth papillae are the longest (9 µm long). The papillae formula is 1 + 1 + 1/3 + 3 + ph. Phasmids are small, located at the base of the ninth papilla. The posterior end of the bursa has a slight indentation on either side of a thready-like tail that measures 13.5 (7.8–19.5) µm long (Figure 1H and Figure 2M).

Infective juveniles are straight when heated, weak, and slender. The stoma is long and narrow. The labial region is rounded, with an asymmetrically pronounced cuticular ridge on the terminal outer surface of the head. Amphidial openings are located more posteriorly than in adults. The stoma and pharynx have the same morphology as in adults (Figure 1L). Pharyngeal procorpus is elongated and slightly thickened. The excretory duct is at the level of the oval basal bulb. The anal canal is blind, not opening outside. The tail is conical, elongated, ending in a pointed tip.

### 3.2. PCA

**Figure 3:**
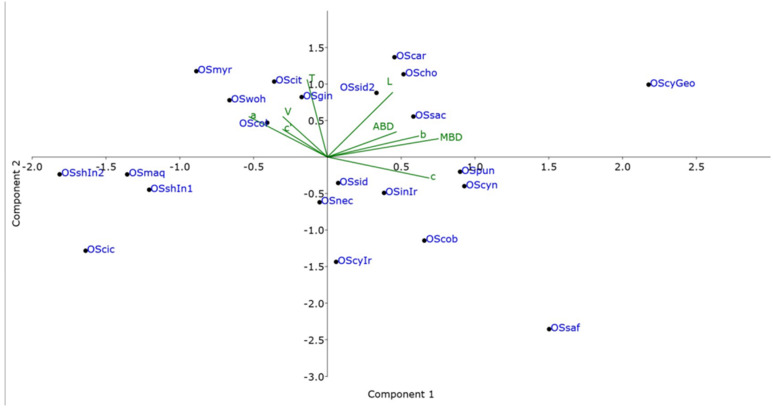
Biplot of Principal Component Analysis (PCA) of the *Oscheius* species. OSaf = *Oscheius safricana*; OScyn = *O. cynodonti*; OScob = *O. cobbi*; OSpun = *O. punctata*; OScyGeo = *O. cyrus* from Georgia; OSsac: *O. sacchari*; OSgin = *O. gingeri*; OSwoh = *O. wolgemuthi*; OScit = *O. citri*; Oscar = *O. carolinensis*; OSchi = *O. chogminensis*; OSsid, OSsid2 = *O. siddiqii*; OScol: *O. colombianus*; OSnec = *O. necromenus*; OScic = *O. ciceri*; OSmyr = *O. myriophulus;* OSshIn1, OSshIn2 = *O. shamimi*; and OSmaq = *O. maqbooli.* L: body length; a: body length/maximum body width; b: body length/pharyngeal length; c: body length/tail length; c′: tail length/body width at anus; V%: (distance from anterior end to vulva/body length) × 100; T: tail length; ABD: anal body diameter; MBD: median body diameter.

Principal Component Analysis (PCA) between nematodes isolated in this study and nematodes of other closely related species was conducted to evaluate morphometric variation between *Oscheius* species. The results of the PCA are represented graphically in Cartesian plots (Figure 3) in which isolates of the *Oscheius* species were projected on the plane of the x- and y-axes, respectively, as pairwise combinations of components PC1 and PC2. An accumulated variability of 57.32% total variance was detected in detail, and the contribution of PC1 was 32.5%, 25.1% for PC2 and 15.13% for PC3. The loading factors for each character were used to interpret the biological meaning of the factors. The body diameter exhibited the highest coefficient of correlation (r = 0.493), and the c ratio (r = 0.452) and b ratio (r = 0.406) were responsible for the significant variability of PC1. This component was associated with the general nematode size. Regarding PC2, almost all characters showed a positive correlation except for the c ratio (r = −0.165). PC2 is mainly dominated by the positive correlation for the tail length (r = 0.610) as well as a high positive correlation for the body length. PC3 was mainly dominated by a high positive correlation for anal body diameter (r = −0.645), b (r = −0.425), and c’ ratio (r = −0.365). Specimens of each species were not casually arranged, and a widespread spatial separation among *Oscheius* species was observed. This spatial separation was mainly dominated by the PC1 grouping the species according to the body dimensions. Some nematode isolates grouped together indicated intraspecific morphological variations across nematode species of this genus. *Oscheius cyrus* from Georgia shows consistent differences in body dimensions (length and maximum body diameter), placing far in the chart from the original description and from other similar species.

### 3.3. Entomopathogenicity Test

An entomopathogenicity test was performed, exposing the fifth instar larvae of *G. mellonella* and *T. molitor* larvae (IV, V, VI stages) to the Georgian population of *O. cyrus*. Insect mortality was assessed every 24 h. Nematodes infecting *Galleria* larvae caused 63.5% mortality within 5 days of inoculation, and infective juveniles emerged from cadavers within 72 h after host death. The highest and fastest mortality (100%) was observed in *T. molitor* larvae after 48 h of inoculation.

### 3.4. Diagnosis and Relationships

The Georgian population of *O. cyrus* is morphologically related to the *O. cyrus* type population [24], with differences concerning the male presence (vs. absent in original description), a larger female body (mean value of 1816 vs. 1193 μm) and lateral fields with eight (vs. six) lines. Such differences could be related to different geographical areas and host species.

*Oscheius* species, both within ‘*Insectivorus*’ and ‘*Dolichura*’ groups show a rather conserved morphology. *Oscheius cyrus* shows close similarities with a few species within the *Insectivorus* group, such as *O. shamimi* [48], *O. carolinensis* [4], and *O. colombiana* [49].

It can also be separated from *O. shamimi* for larger body size in male and female (1019–1341 and 1591–2329 vs. 913–1083 and 951–1255 μm, respectively), longer spicules (39–54 vs. 43–50 μm), and gubernaculum (18–28 vs. 15–18 μm). *Oscheius cyrus* can also be distinguish from *O. carolinensis*, differing in the longer female body length and smaller male body length (1591–2329 vs. 1360–2420 and 1019–1341 vs. 1000–2000 µm, respectively), lower female “a” and male “c” ratios (12.9–18.5 vs. 14.9–23.2 and 17.0–22.9 vs. 32.3 ± 8.4, respectively), fewer lateral lines (4 lines), smaller spicule (48 ± 4 vs. 65 ± 8.9 μm), wider female body diameter and narrower male body diameter (136 ± 9 vs. 95.7 ± 13.6 and 67 ± 8 vs. 73 ± 9.8 μm, respectively).

Compared to *O. colombiana, O. cyrus* from Georgia differs by the longer body of female (1591–2329 vs. 923–1805 μm), higher “c” ratios of female and male, smaller spicule size (39–54 vs. 42–68 μm), fewer lateral lines (8 vs. 4 lines). Compared with populations of *O*. *chongmingensis* [5,50], this species differs in having a longer female body (1591–2329 vs. 1313–2182 µm), a higher female and a smaller male “c” ratio range (13.0–20.5 vs. 8–13 and 17.0–22.9 vs. 18–29 µm, respectively).

### 3.5. Molecular Characterization

PCR amplification of the ITS, D2–D3 expansion domains of the 28S rRNA gene, the partial 18S rRNA gene and the mitochondrial COI of *O. cyrus* produced single products of 852 bp, 642 bp, 1173 bp and 210 bp, respectively, in *H. bacteriophora* of 850 bp, 597 bp, 1595 bp and 220 bp, respectively. Regarding to *O. insectivorus* the D2–D3 expansion domains of the 28S rRNA gene and ITS produced amplicons of 793 bp and 577 bp, respectively.

A BLAST (version 1.30+) search at NCBI of 18S rRNA gene of *O. cyrus* showed 99% identity with a shortest 18S sequence of *O. cyrus* (OM502416; 6 bp different, 2 gaps), and with *O. myriophilus* (20–21 nt different; 3 gaps), *O. colombianus* and *O. insectivorus* (31–32 nt; 3–7 gaps), and *O. citri* (41 nt; 3 gaps). A BLAST search at NCBI of ITS of *O. cyrus* showed a 100% identity (0–3 nt) with *O. cyrus* from Iran and other unidentified *Oscheius* species. A BLAST search at NCBI of D2–D3 expansion domains showed identity with *O. cyrus*, *O. necromenus*, *O. chongmingensis* and unidentified species of *Oscheius*.

Two individual COI amplicons of *O. cyrus* were cloned and four clones were sequenced. A BLAST search at NCBI of COI of O. *cyrus* showed that no identical sequences were present in the database, the closest sequences were those of *O. onirici* and *O. tipulae* with 93% similarity. The intrapopulation sequence diversity was very low, from 0 to 1 nucleotide, at different positions with 4 transitions, 2 A/G and 2 T/C. Pairwise divergences among *Oscheius* COI sequences, including those present in the database, ranged between 0 and 136 bp. Nucleotide sequences were also converted into amino acid sequences and no stop codons or frame shift mutation were present. The amino acid sequence analysis revealed that interspecific amino acid variations among *Oscheius* spp. varied between 0 and 2 amino acids, while with *H. bacteriophora* between 5 and 9 amino acids.

A BLAST search at NCBI of 18S rRNA gene of *H. bacteriophora* showed 99% identity (9–11 nucleotides different) with the corresponding sequences of *H. bacteriophora.* A BLAST search at NCBI of D2–D3 expansion domains showed 99–100% identity (0–6 nt different) with the corresponding region in the database. BLAST search at NCBI of ITS of *H. bacteriophora* showed 100% identity (0–3 nt different) with the corresponding sequences of *H. bacteriophora* in the database.

Three individual COI amplicons of *H. bacteriophora* were cloned and five clones were sequenced. Intrapopulation sequence variability was very low (2 nt different). Blast search revealed 98–99% identity with the corresponding sequences in the database with a range of nucleotide variability between 0 and 11 nt. Pairwise divergences among *H. bacteriophora* COI sequences, including those present in the database, were 0–2.

The D2–D3 region of *O. insectivorus* showed 100% (0–6 nt different) identity with all populations of *O. insectivorus* and *O. shamimi* (MN381940) in GenBank. The *O. shamimi* (MN381940) sequence was identified by [51] as *O. insectivorus,* thus confirming that our population is *O. insectivorus*. BLAST search at NCBI of ITS of the same population showed 100% identity with *O. shamimi* sequences as ITS of *O. insectivorus* is not yet sequenced, thus suggesting a possible misidentification of *O. shamimi* from Iran.

### 3.6. Phylogenetic Analyses

**Figure 4:**
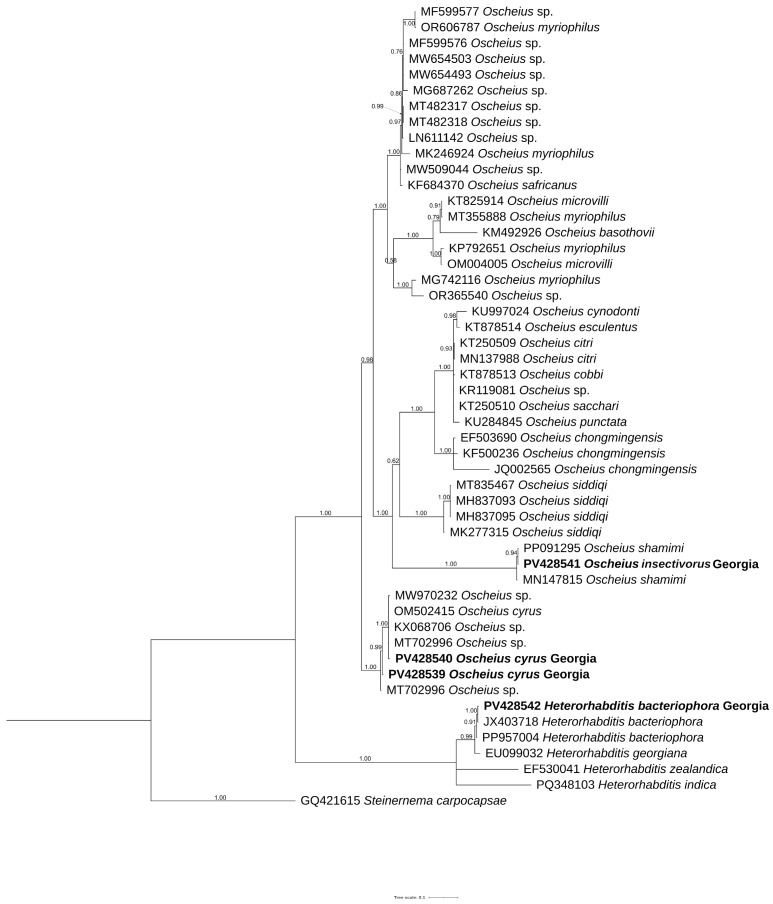
Phylogenetic relationships of *Oscheius cyrus*, *Oscheius insectivorus,* and *Heterorhabditis bacteriophora* from Georgia along with *Oscheius* species. Bayesian 50% majority rule consensus tree as inferred from ITS inferred from symmetrical with gamma distribution (SYM + G) model. Posterior probability values exceeding 50% are given on appropriate clades. Newly obtained sequences in this study are shown in bold.

The new sequences, obtained in the present study, of ITS, D2–D3 expansion domains, 18S rRNA gene and mitochondrial COI were aligned with the corresponding sequences present in the database using Clustal Omega parameters. The ITS phylogenetic tree (Figure 4) included 48 sequences belonging to *Insectivorus*-group and showed different subgroupings. The two sequences of *O. cyrus* formed a well-supported subgroup with *O. cyrus* and other unidentified sequences. *Oscheius insectivorus* grouped with high support with sequences of *O. shamimi*, while *H. bacteriophora* grouped with high support with *H. bacteriophora* and *Heterorhabditis* spp.

**Figure 5:**
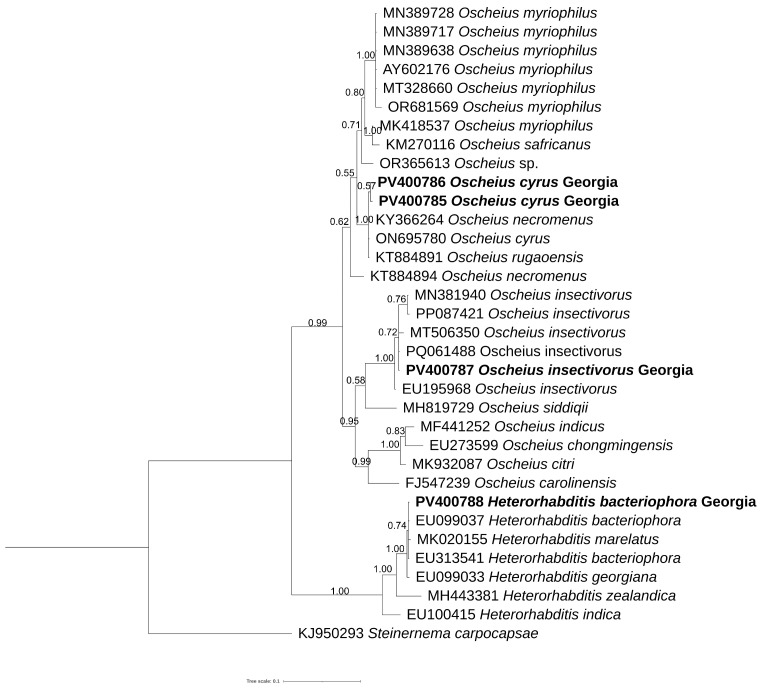
Phylogenetic relationships of *Oscheius cyrus*, *Oscheius insectivorus*, and *Heterorhabditis bacteriophora* from Georgia along with *Oscheius* species. Bayesian 50% majority rule consensus tree as inferred from D2 and D3 expansion domains of 28S rRNA sequence alignment under the General Time-Reversible with gamma distribution (GTR + G) model. Posterior probability values exceeding 50% are given on appropriate clades. Newly obtained sequences in this study are shown in bold.

The D2–D3 tree (Figure 5) showed different subgroupings; one of these contained *O. cyrus* from Iran along with our sequences from Georgia, *O. necromenus* and *O. rugaoensis*, while *O. insectivorus* from Georgia was grouped with all *O. insectivorus* sequences and confirmed that *O. shamimi* (MN381940) was misidentified.

**Figure 6:**
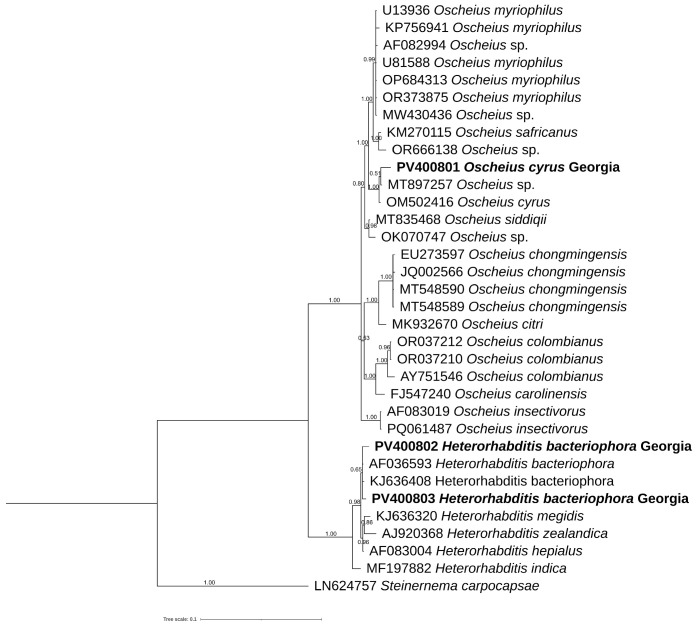
Bayesian tree inferred under the Hasegawa–Kishino–Yano with gamma distribution (HKY + G) model from 18S-rRNA gene sequences of *Oscheius* species. Posterior probability values exceeding 50% are given on appropriate clades. The studied population and newly obtained sequences in this study are indicated in bold text.

Figure 6 shows a phylogenetic tree of the partial 18S rRNA gene in which *O. cyrus* grouped with *O. cyrus* and an undescribed *Oscheius* isolates, while the sequences of *H. bacteriophora* from Georgia with the corresponding ones.

## 4. Discussion

The present study reports on the occurrence of *O. cyrus* and *O. insectivorus* along with *H. bacteriophora* in soil samples recovered from Georgia, confirming Georgia as a hot spot site for entomopathogenic nematodes. The three nematode species were isolated by using the *Galleria* trap method from soil samples.

The identification at morphological level of *Oscheius* spp. is difficult as species belonging to *Oscheius* genus show very conserved gross morphology, causing misidentification also at the sequence level in the literature and in the GenBank database. The incorrect identification negatively influences both biodiversity and evolutionary history of this genus. Recently, new descriptions of *Oscheius* spp. have demonstrated the existence of inaccuracies of drawings and overlapping measurements among several *Oscheius* species [9,51,52]. Thus, in our study, the abundant population of *O*. *cyrus* from hazelnut orchard was characterized by combining morphological, PCA, molecular, and phylogenetic analyses, while *O*. *insectivorus* and *H. bacteriophora* were characterized using sequencing and phylogenetic analyses.

Morphologically, the *Oscheius* population from hazelnut is very close to *O. cyrus* from Iran with little differences (Table 1). PCA analyses, instead, revealed that *O. cyrus* from Georgia in terms of gross morphology clustered separately from *O. cyrus* from Iran, suggesting a potential occurrence of a new *Oscheius* species (Figure 3). But our phylogenetic reconstructions using ITS, D2–D3, and 18S sequences showed that *Oscheius* from Georgia always grouped with *O. cyrus* and other unidentified *Oscheius* spp., thus confirming the occurrence of *O. cyrus* in Georgia hazelnut (Figure 4, Figure 5 and Figure 6). The morphological differences could be due to different geographical origin and host. In the D2–D3 phylogenetic tree (Figure 5), *O. insectivorus* from Georgia subgrouped with all isolates of *O. insectivorus* and *O. shamimi* (MN381940) from GenBank. The sequence of *O. shamimi* (MN381940) was already reported by [51] as misidentification and thus we corrected in *O. insectivorus*. In the ITS phylogenetic tree (Figure 4), *O. insectivorus* from Georgia subgrouped with isolates of *O. shamimi* (PP091295 and MN147815) as no ITS sequences of *O. insectivorus* are present in the database. These results confirm the occurrence of *O. insectivorus* in Georgia. Furthermore, the study of [51] showed that the ITS sequence of *O. shamimi* (MK277315) grouped with high support with *O. siddiqi* isolates, suggesting that this sequence of *O. shamimi* (MK277315) must be considered O. *siddiqi*, and a redescription of *O. shamimi* is urgently needed to correct the corresponding sequences in GenBank and to reduce misidentification.

In addition, in both ITS and D2–D3 phylogenetic trees, *O. safricanus* and *O. basothovii* showed the closest relationships with *O. myriophilus.* Because of the poor quality of morphological figures, they cannot be distinguished as the same species (Figure 4 and Figure 5).

The accurate identification of *Oscheius* species is very important as several of them can be used as biological control agents since different species exhibited different adaptations and behavior [53].

## 5. Conclusions

Our study highlights the importance of identifying nematode species by combining morphological, molecular, and phylogenetic analyses, and of correcting misidentified sequences in GenBank. In the present study, two species, O. cyrus and O. insectivorus, were recovered in Georgia for the first time, increasing their potential to be tested in controlling endemic agricultural pests.

## Figures and Tables

**Table 1:** Morphometrics of female and male of *Oscheius cyrus* from Georgia compared with measurements of other *Oscheius* species from previous studies. All measurements are expressed in µm, in the form: mean ± s.d.(range). L: body length; a: body length/maximum body width; b: body length/pharyngeal length; c: body length/tail length; c’: tail length/body width at anus; V%: (distance from anterior end to vulva/body length) × 100; - =: characters absent or not measured.

Ratios and Character	*Oscheius cyrus*		*O. cyrus*	*O. shamimi*		*O. carolinensis*		*O. colombiana*		*O. chongmingensis*	
	This Work		[24]	[48]		[4]		[49]		[5]	
	Georgia		Iran	India		USA		Colombia		China	
	MALE	FEMALE	FEMALE	MALE	FEMALE	MALE	FEMALE	MALE	FEMALE	MALE	FEMALE
n	20	20	10	10	10	20	20	20	20	20	20
L	1149 ± 91 (1019–1341)	1816 ± 184 (1591–2329)	1193 ± 64 (1097–1293)	994.3 ± 56 (913–1083)	1092 ± 104 (951–1255)	1499 ± 283 (1000–2000)	1728 ± 265 (1360–2420)	915 ± 186 (665–1163)	1288 ± 309 (923–1805)	1015 ± 143 (848–1414)	1684 ± 276 (1313–2182)
*a*	17.2 ± 2.3 (13.1–22.6)	15.5 ± 1 (12.9–18.5)	15.3 ± 3.2 (10.5–19.6)	20.3 ± 1.1 (19–22)	19.7 ± 2.0 (15.4–24.4)	20.4 ± 2.5 (15.8–24.3)	18.2 ± 2.4 (14.9–23.2)	18 ± 2.2 (16–29)	17 ± 1.9 (15–19)	20 ± 1.8 (17–25)	18 ± 1.7 (14–21)
*b*	6.2 ± 0.4 (5.4–7.0)	8.5 ± 0.9 (7.0–10.2)	6.8 ± 1.0 (5.8–8.9)	5.91 ± 0.3 (5.4–6.3)	5.7 ± 0.4 (5.1–6.5)	7.0 ± 1.2 (4.9–8.9)	7.0 ± 1.0 (5.4–9.5)	4.9 ± 0.9 (3.9–5.4)	6.5 ± 1.4 (5.2–8.0)	5 ± 0.5 (4.3–6.6)	6.9 ± 0.9 (5.6–9.0)
*c*	19.9 ± 2 (17.0–22.9)	16.0 ± 2 (13.0–20.5)	13.3 ± 1.0 (12.0–14.5)	34.7 ± 3.2 (30.9–39.6)	8.8 ± 0.6 (7.9–9.8)	32.3 ± 8.4 (18.9–50)	11.0 ± 2.6 (8.4–17.8)	14.5 ± 1.5 (13–16)	9.2 ± 1.1 (8.3–10.0)	23 ± 2.7 (18–29)	11 ± 1.8 (8–13)
*c’*	1.5 ± 0.4 (1.2–1.7)	4.7 ± 0.3 (4–5.8)	4.7 ± 0.9 (3.8–5.9)	1.1 ± 0.2 (0.9–1.5)	5.7 ± 0.76 (4.4–6.9)	1.4 ± 0.3 (1.0–2.3)	4.4 ± 0.9 (3.2–6.1)	2.4 ± 0.1 (2.1–3.5)	4.5 ± 0.1 (3.5–5)	1.5 ± 0.1 (1.2–1.8)	4.2 ± 0.5 (3.1–4.8)
*V%*	-	49.0 ±1 (47.1–51.0)	49.8 ± 1.0 (49–50)	-	48.2 ± 1.3 (46.9–51.2)	-	50.3 ± 1.8 (47.6–55.6)	-	51 ± 3 (47–57)	-	49 ± 2.2 (44–52)
Maximum body diameter	67 ± 8 (52–78)	136 ± 9 (106–143)	80.4 ± 20.1 (42–115)	48.1 ± 3.5 (42–54)	57.4 ± 8.7 (47–80)	73 ± 9.8 (52–89)	95.7 ± 13.6 (67–123)	49 ± 16 (23–72)	81.5 ± 19.5 (49–106)	50 ± 7.9 (43–74)	94 ± 16 (74–141)
Tail lenght	57 ± 5 (49–67)	132 ± 14 (96–153)	77.6 ± 19.0 (45–100)	27.7 ± 3.9 (23–35)	121.5 ± 6.6 (110–130)	48 ± 8.6 (32–64)	160 ± 26.6 (108–206)	63.5 ± 7 (51–70.5)	140 ± 18 (110–167)	45 ± 3.7 (38–56)	157 ± 20 (121–188)
Anal Body width	30 ± 5 (23–39)	44 ± 6 (41–54)	19.4 ± 3.1 (15–23)	24.8 ± 0.9 (24–27)	21 ± 3.8 (17–28)	34 ± 4.6 (25–40)	36 ± 3.6 (28–43)	26 ± 5 (15–32)	31 ± 5 (22–38)	31 ± 2.6 (25–38)	37 ± 2.9 (31–43)
Spicule length	48 ± 4 (39–54)	-	-	46.1 ± 2.1 (43–50)	-	65 ± 8.9 (50–81)	-	56 ± 9 (42–68)	-	47 ± 5.1 (40–59)	-
Gubernaculum length	22 ± 3 (18–28)	-	-	16.6 ± 1.2 (15–18)	–	29.4 ± 4.8 (20–35)	-	20 ± 2 (16–24)	-	25 ± 4.2 (19–32)	-
Lateral field (incisures)	8		6	6		4		4		6	
Bursa	Leptoderan	-	-	Leptoderan	-	Leptoderan	-	Leptoderan	-	Pseudo-peloderan	-
Papillae	1 ± 1 ± 1/3 ± 3 ± ph	-	-	1 ± 1 ± 1/3 ± 3 ± ph	-	1 ± 1 ± 1/3 ± 3 ± ph	-	1 ± 1 ± 1/3 ± 3 ± ph	-	1 ± 2 ± 3/3	
host (plant)	*Corylus avellana*		Forest			Vermicompost				Soil	
host (insect)				*Scarites indus* (Coleoptera: Carabidae) Olivier 1795.				*Cyrtomenus bergi* (Hemiptera: Cydnidae) Fröschner			

## Data Availability

Data are contained within this article.
